# Mirage or long-awaited oasis: reinvigorating T-cell responses in pancreatic cancer

**DOI:** 10.1136/jitc-2020-001100

**Published:** 2020-08-25

**Authors:** Michael Brandon Ware, Bassel F El-Rayes, Gregory B Lesinski

**Affiliations:** Hematology and Oncology, Emory University Winship Cancer Institute, Atlanta, Georgia, USA

**Keywords:** T-lymphocytes, immunomodulation, immunotherapy, tumor escape

## Abstract

Pancreatic ductal adenocarcinoma (PDAC) is plagued by a dismal 5-year survival rate, early onset of metastasis and limited efficacy of systemic therapies. This scenario highlights the need to fervently pursue novel therapeutic strategies to treat this disease. Recent research has uncovered complicated dynamics within the tumor microenvironment (TME) of PDAC. An abundant stroma provides a framework for interactions between cancer-associated fibroblasts, suppressive myeloid cells and regulatory lymphocytes, which together create an inhospitable environment for adaptive immune responses. This accounts for the poor infiltration and exhausted phenotypes of effector T cells within pancreatic tumors. Innovative studies in genetically engineered mouse models have established that with appropriate pharmacological modulation of suppressive elements in the TME, T cells can be prompted to regress pancreatic tumors. In light of this knowledge, innovative combinatorial strategies involving immunotherapy and targeted therapies working in concert are rapidly emerging. This review will highlight recent advances in the field related to immune suppression in PDAC, emerging preclinical data and rationale for ongoing immunotherapy clinical trials. In particular, we draw attention to foundational findings involving T-cell activity in PDAC and encourage development of novel therapeutics to improve T-cell responses in this challenging disease.

## Immunosuppression and a harsh stromal microenvironment drive therapeutic resistance in pancreatic ductal adenocarcinoma (PDAC)

PDAC is a devastating malignancy in dire need of novel therapies. Single-agent immune checkpoint blockade has historically elicited almost no response in PDAC, outside of rare patients harboring genetic alterations impacting microsatellite instability.^1–3^ Similarly, vaccine or cellular therapies in PDAC demonstrate only modest effects, although these modalities remain in early stages.^4–6^ Many clinical challenges arise from rapid progression of PDAC, often presenting as metastatic disease.^7^ It is hypothesized that the aggressive nature of this disease and failure of many therapies can be attributed to dominant immunosuppressive features in the PDAC tumor microenvironment (TME).

The TME of PDAC has unique characteristics in comparison to other tumor types. It is dominated by a fibrotic and desmoplastic stroma containing diverse populations of cancer-associated fibroblasts and immunosuppressive myeloid cells, with sparse T-cell infiltration.^8–10^ This PDAC-associated stroma, often composing up to 90% of tumors by volume, presents a dynamic and insurmountable barrier to immunotherapy.^9 11 12^ In recent years, advanced murine models of PDAC and forward-thinking approaches have unveiled important mechanisms of immune suppression in PDAC. Additionally, our understanding of how effective antitumor responses can be generated in PDAC is advancing with a cautious optimism for successful application of immunotherapy in this deadly cancer. Here we describe recent findings related to immune suppression in PDAC, highlighting successful advances, and priority areas for future research and discovery.

## Immune privilege of pancreatic cancer

T cells can intrinsically promote antitumor responses in coordination with a diverse array of cell types. Recent advances in immunohistochemistry (IHC) and microscopy, in addition to flow cytometry, have allowed for more precise quantification of immune infiltration in PDAC and revealed pancreatic tumors are largely devoid of effector T-cell infiltration and immune privilege.^8–10^ An eloquent study using multispectral IHC^9^ compared localization of T-cell and myeloid subsets in the stromal and tumor compartments in both melanoma and pancreatic cancer. The rationale for parallel analysis of these distinct tumor types was to compare differences in the infiltration of T cells and response to immune therapy. While comparing tissue of pancreatic cancer cases with poor or positive response to immunotherapy regimens would be preferential, the lack of immune response to PDAC necessitated this approach of comparing to immune responsive melanoma. Analysis of PDAC tissue revealed relatively few T-cell infiltrates as marked by CD3 and CD4 or CD8 staining compared with melanoma.^9^ This is certainly troublesome, since increased infiltration of CD4^+^ and CD8^+^ T cells in tumors is consistently associated with increased survival in patients.^8 13–16^ This observation parallels immune suppressive features of other tumors, including prostate and breast cancers. Certainly, emerging evidence in these other solid tumors points to a diverse array of complex intracellular mechanisms in the TME mediating T-cell inactivity, including T-regulatory activity and myeloid derived suppressor cell function.^17–25^ While this review focuses on PDAC, many observations described here will likely hold true for other ‘immunologically cold’ tumor types.

### Low mutational burden and poor immunogenicity fails to induce T-cell infiltration

Lack of effector T-cell infiltration in PDAC has been hypothesized to be a product of poor tumor immunogenicity stemming, in part, from lower frequency of neoantigens.^26^ Attempts to directly interrogate the immunogenicity of PDAC have employed sophisticated techniques involving patient tissue and the genetically engineered KPC mouse model (*LSL-Kras*^*G12D/+*^;*LSL-Trp53*^*R172H/+*^;*Pdx-1-Cre*), which recapitulates much of the microenvironment in human PDAC.^27 28^ Impressive efforts have employed novel methods to isolate and sequence neoplastic cells within pancreatic tumors while excluding stromal regions which may have confounded past studies.^29 30^ These reports indicate a complex and highly diverse mutational landscape in PDAC that challenges previous work.^29^ Certainly, recent clinical data from the ‘Know your Tumor’ initiative demonstrated that choice of personalized, targeted therapy based on genomic features can improve outcomes in PDAC.^31^ While PDAC is capable of appropriate antigen stimulation of T cells, these studies indicate release or presentation of antigen may be inhibited or obscured in cases with poor T-cell response.

### Stromal barriers to T-cell infiltration at the margin of pancreatic tumors

**Figure 1:**
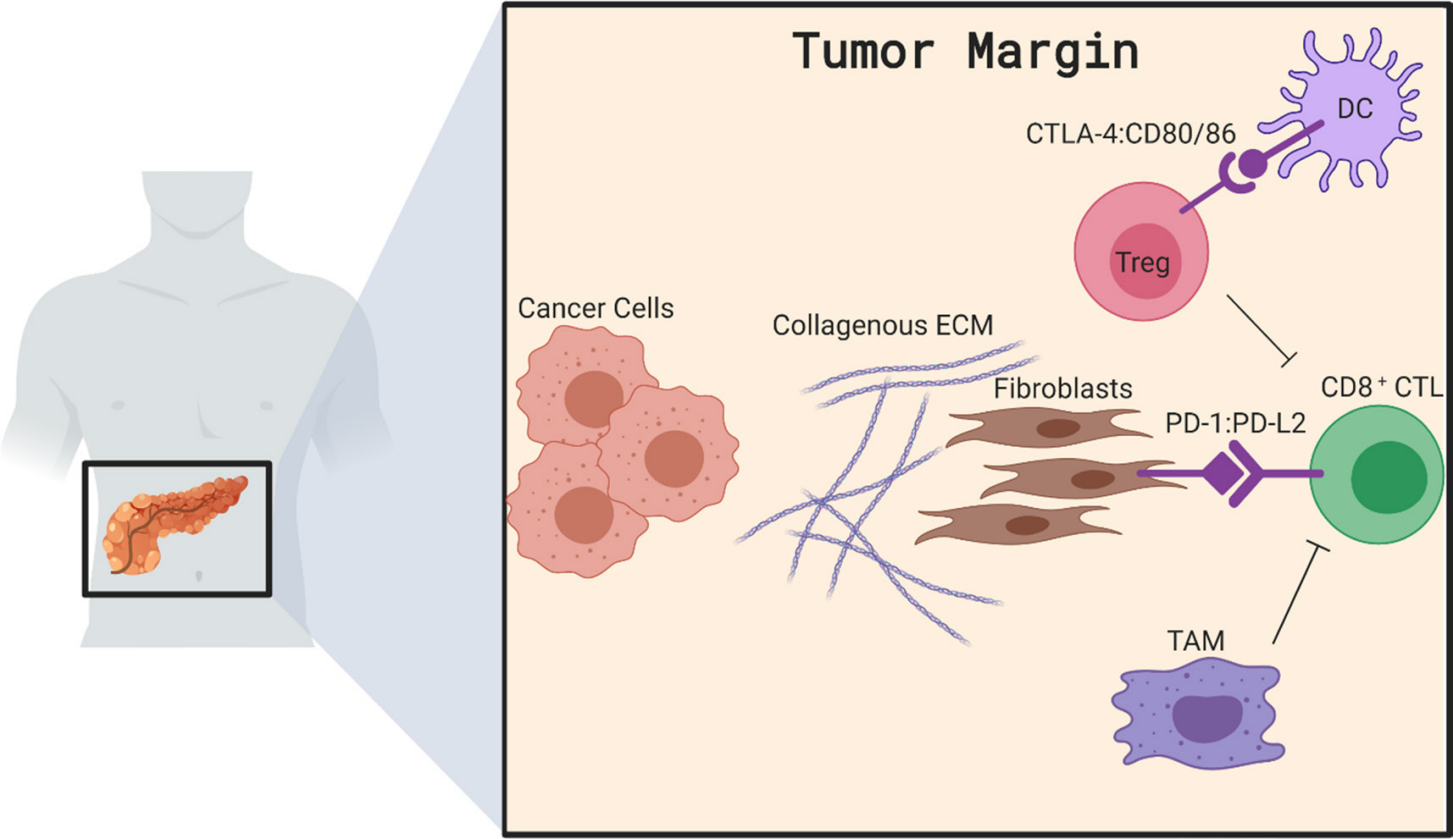
TME of pancreatic tumors encompasses heterogenous cell populations that collectively prevent T-cell infiltration of pancreatic tumors. Here we illustrate T-regulatory cells acting to directly suppress CD8 CTLs while also blocking T-cell priming by occupying dendritic cells. Multiple populations of fibroblasts produce extracellular matrix to drive fibrosis or express PD-L2, which sequesters T cells, while altering the balance of cytokines. TAMs also play a role in sequestering CD8^+^ CTLs at the tumor margin to prevent efficient infiltration. Together these TME interactions contribute to the immunologically ‘cold’ state of pancreatic tumors. CTL, cytotoxic lymphocyte; PD-L2, programmed death ligand 2; TAM, tumor-associated macrophage; TME, tumor microenvironment; CTLA-4, cytotoxic T-lymphocyte-associated protein 4.

Perhaps the most unique aspect of pancreatic cancer is the overwhelming stroma which shapes the TME. The abundant stroma associated with PDAC has been long hypothesized to physically restrain T-cell and therapeutic drugs or antibodies due to the collagen, fibronectin and other extracellular matrix (ECM) components secreted by fibroblasts and cancer cells.^32^ However, research by two separate groups demonstrated no significant relationship between thickness of the desmoplasia, fibrotic content in the TME or presence of cancer-associated fibroblasts with the exclusion of infiltrating T cells from neoplastic lesions.^8 33^ While the stroma represents a barrier for T-cell infiltration into PDAC, these studies indicate this exclusion may occur through mechanisms more complicated than only a physical barrier. Indeed, research dissecting individual components of PDAC stroma and associated mechanisms reveals complex immunosuppressive mechanisms involving cancer-associated fibroblasts, T-regulatory cells (Tregs), tumor-associated macrophages (TAMs) and dendritic cells, each of which affect T-cell infiltration into tumors (figure 1).

### Diverse fibroblast populations contribute to immune suppression in PDAC

The fibroblast components of PDAC tumors are riddled by heterogeneity and plasticity. Previous research defined distinct populations of fibroblasts within PDAC possessing inflammatory or myofibroblastic properties termed inflammatory cancer associated fibroblasts (iCAFs) and myofibroblastic cancer associated fibroblasts (myCAFs), respectively.^34^ These cells have potential to modulate tumor growth and stromal composition and may alter immune responses to PDAC by contact-dependent and independent properties.^34^ The inflammatory iCAF subsets are characterized by production of soluble factors, such as interleukin (IL)-6, leukemia inhibitory factor and IL-11, with immune modulatory potential.^34 35^ myCAFs assume a more traditional activated fibroblast phenotype, secreting ECM components such as collagen and fibronectin.^34–36^ Work by Ohlund *et al* elucidated a dynamic interplay between tumor cell-derived interleukin-1-alpha (IL-1α) and transforming growth factor beta (TGFβ) within the stroma that significantly influences cancer associated fibroblast (CAF) fate.^35^ IL-1α from cancer cells polarized directly adjacent CAFs to a myCAF phenotype; however, IL-1α signaling can be disrupted by the presence of TGFβ in more distant stromal regions, promoting the inflammatory profile seen in iCAFs.^35^ Of note, TGFβ activation in the stroma has been linked to infiltration and activity of non-degranulated mast cells, which associate with CAFs, and whose infiltration has been linked with worse overall survival in tissue from previously untreated patients with resectable PDAC.^37–40^

Cross-species sequencing of pancreatic tumors in mice and humans has also revealed the existence of another interesting CAF population with the ability to present antigen.^36^ These antigen-presenting CAFs express both CD74 and major histocompatability complex-II (MHC-II), indicating a propensity to present antigen to CD4^+^ T cells in vivo, potentially resulting in increased activation of CD4^+^ T cells.^36^ The plasticity of these CAF populations and this ‘Jekyll and Hyde’ influence on the immune system present a complicated case for targeting the stroma to mediate immune activation in PDAC. Indeed, past challenges with pharmacological agents targeting stromal pathways such as sonic hedgehog have rightfully tempered enthusiasm for launching into clinical trials without rigorous data.^41^ Furthermore, two key reports have demonstrated that in vivo depletion of fibroblasts in murine models resulted in aggressive progression toward metastatic disease and that degree of stroma was inversely related to clinical outcome.^42 43^ Despite these data, tumors that arose in mice lacking α-SMA^+^ fibroblasts were exquisitely sensitive to immunotherapy, again implying the stroma restrains immune response to PDAC tumors. Taken together, these data indicate consideration of individual CAF subsets is likely necessary in designing approaches to treat PDAC.^42–44^

## Intercellular dynamics mediating T-cell exclusion from PDAC

### Cancer-associated fibroblasts have heterogeneous effects on T-cell activation

More recently, checkpoint-mediated interactions between CAFs and pancreatic cancer cells (PCCs) have been implicated as a mechanism by which T cells are trapped and killed or inactivated in the PDAC stroma.^45^ PDAC-associated CAFs display higher expression of programmed death ligand 1 (PD-L1) and programmed death ligand 2 than normal fibroblasts, with the latter more highly expressed. In vitro experiments demonstrate the ability of CAFs to upregulate programmed cell death protein 1 (PD-1), cytotoxic T-lymphocyte-associated protein 4 (CTLA-4), and T-cell immunoglobulin and mucin domain-containing protein 3 on both CD4^+^ and CD8^+^ T cells, as well as lymphocyte-activation gene 3 on CD4^+^ T cells. This phenotypical shift eventually leads to decreased T-cell proliferation. Alternatively, fibroblast populations in the TME of PDAC can control immunity through contact-independent mechanisms such as secretion of cytokines and chemokines. In addition to secretory factors discussed previously, other investigations indicate a role for fibroblast-derived CXCL12 in facilitating T-cell exclusion in PDAC.^46^ Feig *et al* found CXCL12 from fibroblasts was responsible for excluding T cells in PDAC and mediating failure of both αPD-L1 and αCTLA-4 therapy.^46^ These data have led to clinical trials blocking the receptor for CXCL12 (CXCR4) with the Food and Drug Administration-approved drug plerixafor (NCT02179970). These results highlight the numerous complementary aspects of the PDAC stroma that drive T -cell exclusion from PDAC.

### Duality of lymphocytes within the context of antitumor immunity

**Figure 2:**
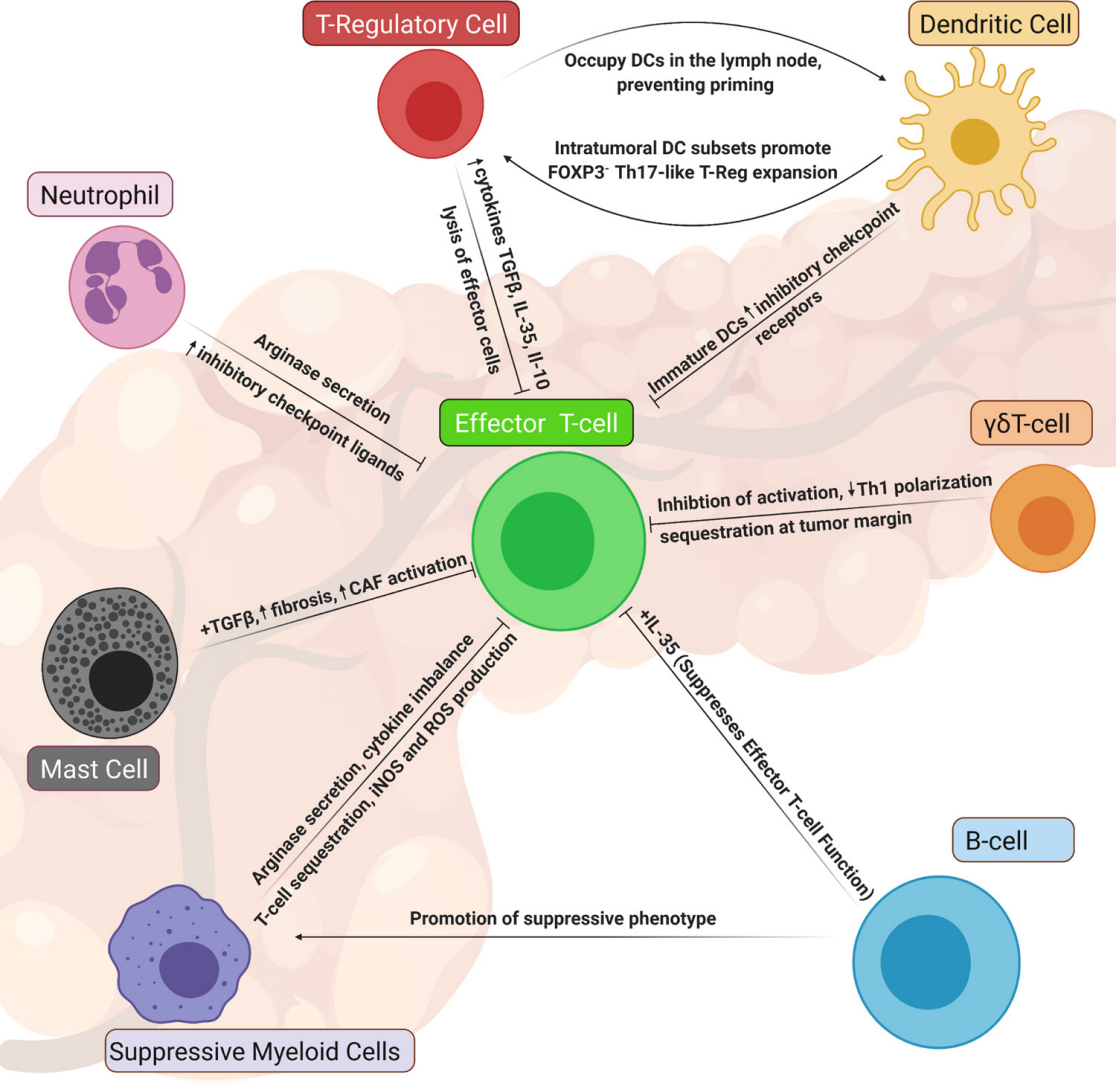
Dismal T-cell responses observed in pancreatic cancer can be attributed, in part, to a multitude of inflammatory monocytes and suppressive lymphocytes within the tumor microenvironment of pancreatic tumors. Here, we highlight populations of immune suppressive cells in PDAC that have been understudied yet have been shown to directly and indirectly suppress effector T cells in PDAC. notably, many of the mechanisms highlighted here involve soluble mediators, such as chemokines, cytokines, growth factors and reactive nitrogen species and ROS. These cellular populations should be more commonly considered as we seek to develop novel therapeutic strategies to reinvigorate T-cell activity in PDAC. DC, dendritic cell; IL, interleukin; PDAC, pancreatic ductal adenocarcinoma; ROS, reactive oxygen species; TGFβ, transforming growth factor beta; cancer associated fibroblast, CAF; inducible Nitrous Oxide Synthase iNOS; myeloid-derived suppressor cell, MDSC.

Interestingly, immunosuppressive Tregs and B cells with regulatory properties can localize to stromal areas of PDAC, rather than within foci of adenocarcinoma.^9 47–50^ These Tregs are most often characterized as CD4-positive, with high expression of the IL-2 receptor CD25 and the transcription factor Forkhead Box P3 (FOXP3). Definitive histological detection of these cells in tissue is challenging, and often their characterization omits CD25 for technical simplicity.^51 52^ Like effector CD4^+^ or CD8^+^ T cells, Tregs preferentially localize to stroma, rather than tumor foci in PDAC, but can be found in uninvolved and tumor compartments in equal proportion.^9 53^ However, the central location for the inhibitory action of these cells may be in peritumoral lymph nodes associated with PDAC. Indeed this is where the majority of Tregs in tumor-bearing mice are found.^53^ This research also revealed CTLA4/CD80 interactions between Tregs and dendritic cells (DCs) as essential molecular mediators of CD4 T-cell exclusion, but the specifics of how CD4 T cells are actually excluded as a result of these interactions are only now becoming clear. A novel observation by Jang *et al* describes prolonged interactions between Tregs and DCs in PDAC, demonstrating the ability for Tregs to outcompete CD8^+^ T cells and limit CD8^+^ T-cell interactions with DCs^54^ (figure 2). In this manner, Tregs limit T-cell priming in the periphery and significantly diminish cytotoxic T-cell responses to PDAC.^53 54^

### Alternative regulatory T cells lacking FOXP3 expression contribute to immune suppression

Naturally occurring Tregs express the transcription factor FOXP3^51 55–57^; however, CD4^+^ Tregs without canonical FOXP3 expression can also repress immune responses.^58 59^ In fact, a CD4^+^ Treg subset positive for IL-10 and IL-17 and negative for FOXP3 was identified in murine PDAC models.^60^ These FOXP3^−^ Tregs promote tumor progression and have a similar phenotype to type I regulatory (Tr1) cells that develop from mature antigen-stimulated CD4^+^ T cells.^60 61^ Tr1 cells, identified over 30 years ago in patients, characteristically secrete large amounts of IL-10 and can negatively impact antigen-presenting myeloid cells.^62 63^ While natural Tregs traditionally develop in the thymus from naïve cells, Tr1 cells can be altered and differentiated in the TME of both mouse and human hosts, identified by their expression of CD49 and LAG3.^60 61 64^ A recent study by Barilla *et al* describes the influence of specialized DC subsets in skewing of CD4^+^ T cells to this Tr1 phenotype and the protumorigenic effect of this interaction^60^ (figure 2). Additionally, CD4^+^ T cells exposed to a suppressive DC subset from the PDAC TME shifted to a Th17 phenotype, including a population of Th17-like cells expressing FOXP3.^60^ Thus, DC subsets have the capacity to induce multiple regulatory T-cell subsets which suppress immune responses.^60^ The expansion of these cells from antigen-experienced CD4^+^ T cells also diminishes potential helper cells within the PDAC TME. Interestingly, DCs isolated from pancreata or spleens of naïve mice or from spleens of PDAC tumor-bearing mice do not have the same abilities, indicating PDAC exerts a unique influence over DCs in the TME.^60^ These data highlight the complex interactions mediating the presence of effector T-cell populations within the TME.

### PDAC-associated B cells limit cytotoxic T-cell activity

B lymphocytes, or B cells, can have immunosuppressive activity in several tumor types.^47–50^ B cells can associate with CD8^+^ T cells in both murine PDAC models and pancreatic intraepithelial neoplasias (PanIN) of patients.^48 50^ Several studies have uncovered B-cell phenotypes in PDAC, and targeting these cells improves immune responses in PDAC.^48–50^ Emerging reports highlight a role for B-cell-derived IL-35, as well as B-cell control of macrophage polarization to a tumor-promoting phenotype. While separate studies have alternatively defined tumor-promoting B-cell subsets, it should be noted that growth of orthotopic pancreatic tumors in B-cell-deficient mice (μ*M*T) was severely diminished.^50^ Further, depletion of B cells in mice with PanIN significantly inhibited progression.^48^ Several pathways such as IL-35 secretion, dynamic fluctuation of hypoxia-inducible factor 1-alpha and Bruton tyrosine kinase activation are potential targets for inhibiting B cells in PDAC^48–50^ (figure 2). Balancing these immune suppressive properties of B cells in PDAC are other strong data demonstrating B cells can cluster in tertiary lymphoid tissues (TLTs). Further, B-cell clustering is significantly correlated with improved T-cell activity in murine models^65^ and more favorable outcomes for patients with PDAC.^66 67^ While TLTs have recently emerged as an interesting feature of potent antitumor responses,^68^ their presence and make-up in PDAC tissues is quite understudied. Indeed, the role of B cells and TLT in PDAC progression deserves further exploration.

### More than M1/M2: complex interactions of TAMs suppress T-cell responses to PDAC

Macrophages represent a sizeable proportion of cells in the PDAC TME and have complex characteristics. Available evidence suggests these cells are either derived from circulating monocytes, or established in the organ during embryonic development.^69^ Through a set of elegant preclinical experiments, the role of these two macrophage lineages in the PDAC TME has been elucidated. These studies revealed embryonically derived, TAMs promote fibrosis and tumor growth, while monocyte-derived TAMs directly influence immune suppression.^69^ Embryonically-derived TAMs are distinguished by expression of colony-stimulating factor one receptor (CSF1R) in mice and CXCR4 in humans, and expand during tumor development.^69^ In contrast to established roles for these TAM subsets, dynamic imaging microscopy showed prolonged interactions between TAMs and CD8^+^ T cells in murine pancreatic tumors.^69^ These interactions were localized to dense stromal regions of tumors, whereby T cells were trapped and prevented from infiltrating tumors.^69^ These data indicate a dual role for embryonically derived TAMs in promoting fibrosis and tumor growth and also preventing the infiltration of cytotoxic T cells into tumors.

In comparison, monocyte-derived TAMs express high levels of MHC-II and are more adept at sampling and presenting antigen. Monocyte-derived TAMs in circulation can infiltrate into pancreatic tumors or tumor-draining lymph nodes, by virtue of interactions with chemokine receptor 2 (CCR2).^24 70–73^ A recent study characterizing extratumoral Ly6C^low^ F4/80^+^ macrophages (monocytic phenotype markers) demonstrates these cells act outside of tumors to drive CD4^+^ T-cell-specific exclusion from PDAC.^74^ Clodronate depletion of macrophages from mice with spontaneously arising PDAC increased infiltration of CD4^+^ T cells into tumors.^74^ As with studies of Tregs, the effects of macrophages on CD4^+^ T-cell exclusion are localized to extratumoral locations, as clodronate had no effect on macrophages in the TME.^74^

Taken together, the available data indicate investigation should extend to regions outside of tumor tissue, to consider how local and distal mechanisms influence immune suppression in PDAC. In addition to T-cell exclusion, there are numerous immune interactions in PDAC mediating suppression of T-cell activation, which we highlight in figure 2.

## Therapeutic approaches to ameliorate T-cell exclusion and inactivation in PDAC

The extent to which T cells are both excluded and suppressed in PDAC may seem disheartening; however, as the obstacles become more well defined, our therapeutic strategies continue to improve in their sophistication. Recent advances have furthered our characterization and understanding of how T cells are inhibited from eliminating pancreatic tumors. A fibrotic and desmoplastic TME, inadequate exposure to tumor-associated antigens and numerous suppressive cells represent areas of investigation. While these factors bear weight on T-cell responses, they also uncover opportunities to dismantle specific aspects of immune suppression. Cellular and targeted agents aimed at blocking cell–cell interactions or crosstalk mediated by soluble factors have promise for re-engaging T-cell responses to PDAC. Here, we discuss a series of select therapeutic strategies being leveraged to advance immunotherapy in PDAC.

### Combinatorial approaches with immune checkpoint inhibition (ICI)

ICI, specifically targeting CTLA-4 or the PD-1/programmed death ligand 1 (PD-L1) axis, has gained traction due to success in several oncological settings. Unfortunately, these drugs have shown little promise as single agents for patients with PDAC.^2 75^ Equipped with new and emerging knowledge of immune suppression in PDAC, many groups are pursuing combinatorial approaches to alleviate immune suppression and enhance ICI in PDAC. These include neutralization of growth factors or cytokines, inhibition of chaperone proteins and kinases, simultaneous blockade of multiple immune checkpoints and a host of others. Many strategies have emanated from encouraging preclinical results into first in human clinical trials in the setting of PDAC (table 1). It is worth noting that the effects of radiation and chemotherapy, which are commonly incorporated into combination therapeutic strategies (table 1), have not been fully characterized with respect to their immune influence. Recent studies addressing the effects of radiation have revealed this approach to control local tumor growth but with detrimental effects on antitumor immunity within the TME. Results from these studies indicate an influx in suppressive macrophages, Tregs and increases in inducible Nitic Oxide Synthase (iNOS) release by cancer-associated fibroblasts that together result in poor T-cell activity and sparse infiltration of pancreatic tumor tissues.^76–78^ In contrast, chemotherapy is hypothesized to increase T-cell priming, which we discuss further briefly, and data from our group and others indeed suggest immune changes elicited by chemotherapy are significant.^76 79^ Thus, considering how immunotherapy approaches can be strategically combined with radiation or chemotherapy will be key in moving forward.

**Table 1:** Ongoing and emerging clinical trials in PDAC using CAR-T therapy and novel combinations with ICI

Interventions	Phases	Locations	NCT number	Status
Viral and vaccine-based therapies				
Pembrolizumab|wild-type reovirus	Phase II	Northwestern University, Chicago, Illinois, USA	NCT03723915	Ongoing
GRT-C903|GRT-R904|nivolumab|ipilimumab	Phase I Phase II	Multicenter	NCT03953235	Ongoing
Cyclophosphamide|nivolumab|ipilimumab|GVAX pancreas vaccine|CRS-207	Phase II	Johns Hopkins SKCCC, Baltimore, Maryland, USA	NCT03190265	Ongoing
Cyclophosphamide|nivolumab|GVAX pancreas vaccine|radiation: SBRT	Phase II	Multicenter	NCT03161379	Ongoing
Epacadostat|pembrolizumab|CRS-207|CY|GVAX	Phase II	The Sidney Kimmel Comprehensive Cancer Center at Johns Hopkins, Baltimore, Maryland, USA	NCT03006302	Recruiting
Cyclophosphamide|GVAX|pembrolizumab|radiation: SBRT	Phase II	The Sidney Kimmel Comprehensive Cancer at Johns Hopkins, Baltimore, Maryland, USA	NCT02648282	Ongoing
Cyclophosphamide|GVAX|pembrolizumab|IMC-CS4	Early phase I	Sidney Kimmel Comprehensive Cancer Center, Baltimore, Maryland, USA	NCT03153410	Recruiting
CAR-T or TIL-based therapies				
Activated CIK and CD3-MUC1 bispecific antibody in treating pancreatic cancer|procedure: cryotherapy	Phase II	Institutional Review Board of Guangzhou Fuda Cancer Hospital, Guangzhou, Guangdong, China	NCT03509298	Ongoing
Anti-MUC1 CAR-pNK cells	Phase I Phase II	PersonGen BioTherapeutics (Suzhou) Co, Ltd, Suzhou, Jiangsu, China	NCT02839954	Unknown status
Anti-MUC1 CAR-T cells	Phase I Phase II	PersonGen Biomedicine (Suzhou) Co, Ltd, Suzhou, Jiangsu, China	NCT02587689	Unknown status
Anti-CEA CAR-T Cells| gemcitabine/nab paclitaxel| NLIR+FU/FA|capecitabine	Phase II Phase III		NCT04037241	Not yet recruiting
multiTAA specific T cells	Phase I Phase II	Baylor Clinic, Houston, Texas, USA|Houston Methodist Hospital, Houston, Texas, USA|Harris Health System, Smith Clinic, Houston, Texas, USA	NCT03192462	Ongoing
BPX-601|rimiducid	Phase I Phase II	Multicenter	NCT02744287	Ongoing
Young TIL|aldesleukin|cyclophosphamide|fludarabine|pembrolizumab (Keytruda)	Phase II	National Institutes of Health Clinical Center, 9000 Rockville Pike, Bethesda, Maryland, USA	NCT01174121	Ongoing
TEW-7197	Phase I Phase II	Samsung Medical Center, Seoul, Republic of Korea	NCT03666832	Ongoing
Pegylated recombinant human hyaluronidase PH20|pembrolizumab	Phase II	M D Anderson Cancer Center, Houston, Texas, USA	NCT04058964	Not yet recruiting
Targetted small mlecule and antibody-based therapies in combination with ICI				
Anti-SEMA4D monoclonal antibody VX15/2503|ipilimumab|nivolumab|procedure: surgery	Phase I	Emory University, Atlanta, Georgia, USA	NCT03373188	Ongoing
APX005M|nivolumab|nab-paclitaxel|gemcitabine	Phase I Phase II	Multicenter	NCT03214250	Active, not recruiting
XL888|pembrolizumab	Phase I	Emory University, Atlanta, Georgia, USA	NCT03095781	Recruiting
Pembrolizumab|defactinib	Phase II	Sidney Kimmel Comprehensive Cancer Center, Baltimore, Maryland, USA	NCT03727880	Ongoing
Antibiotics and pembrolizumab	Phase IV	NYU Langone Health, New York, New York, USA	NCT03891979	Not yet recruiting
ENB003 plus pembrolizumab phase Ib/IIa in solid tumors			NCT04205227	
ENB003|pembrolizumab	Phase I Phase II		NCT04205227	Not yet recruiting
GB1275|nab-paclitaxel and gemcitabine|pembrolizumab	Phase I Phase II	Multicenter	NCT04060342	Ongoing
Pembrolizumab|sonidegib	Phase I	Mayo Clinic in Arizona, Scottsdale, Arizona, United States|Mayo Clinic in Florida, Jacksonville, Florida, United States|Mayo Clinic, Rochester, Minnesota, USA	NCT04007744	Ongoing
XmAb22841|pembrolizumab (Keytruda)	Phase I	Multicenter	NCT03849469	Ongoing
FT500|nivolumab|pembrolizumab|atezolizumab|cyclophosphamide|fludarabine	Phase I	Multicenter	NCT03841110	Ongoing
PEGPH20|pembrolizumab	Phase II	Multicenter	NCT03634332	Ongoing
CPI-006| CPI-006+ciforadenant|CPI-006+pembrolizumab	Phase I	Multicenter	NCT03454451	Ongoing
Pembrolizumab|paricalcitol|placebo	Phase II	Multicenter	NCT03331562	Active, not recruiting
INT230-6|anti-PD-1 antibody|anti-CTLA-4 antibody	Phase I Phase II	Multicenter	NCT03058289	Ongoing
CXCR4 Antagonist BL-8040| Pembrolizumab|Other: Pharmacological Study	Phase II	M D Anderson Cancer Center, Houston, Texas, USA	NCT02907099	Active, not recruiting
Adoptive immunotherapy|aldesleukin|cyclophosphamide|other: laboratory biomarker analysis| pembrolizumab	Phase I	M D Anderson Cancer Center, Houston, Texas, USA	NCT02757391	Active, not recruiting
Pembrolizumab|itacitinib|INCB050465	Phase I	Multicenter	NCT02646748	Active, not recruiting
Pegilodecakin|paclitaxel or docetaxel and carboplatin or cisplatin|FOLFOX (oxaliplatin/leucovorin/5-fluorouracil)|gemcitabine/nab-paclitaxel|capecitabine|pazopanib|pembrolizumab|paclitaxel|nivolumab| gemcitabine/carboplatin	Phase I	Multicenter	NCT02009449	Active, not recruiting
Nivolumab|ipilimumab|tocilizumab|radiation: SBRT	Phase II	Herlev & Gentofte University Hospital, Denmark, Herlev, Denmark	NCT04258150	Ongoing
BT5528|nivolumab	Phase I Phase II	Multicenter	NCT04180371	Ongoing
KRAS peptide vaccine|nivolumab|ipilimumab	Phase I	Sidney Kimmel Comprehensive Cancer Center, Baltimore, Maryland, USA	NCT04117087	Not yet recruiting
Nivolumab|radiation: radiation therapy|TLR9 agonist SD-101	Phase I	University of California Davis Comprehensive Cancer Center, Sacramento, California, USA	NCT04050085	Ongoing
Part 1 TPST-1120|part 2a TPST-1120+nivolumab|part 2b TPST-1120+docetaxel|part 2c TPST-1120+cetuximab|part 3 TPST-1120|part 4a TPST-1120+nivolumab|part 4b TPST-1120+docetaxel|part 4c TPST-1120+cetuximab	Phase I	Multicenter	NCT03829436	Ongoing
Anetumab ravtansine|gemcitabine hydrochloride|ipilimumab|nivolumab	Phase I Phase II	Multicenter	NCT03816358	Ongoing
Nivolumab|tadalafil|oral vancomycin	Phase II	National Institutes of Health Clinical Center, Bethesda, Maryland, USA	NCT03785210	Ongoing
Radiation: SBRT|nivolumab|CCR2/CCR5 dual antagonist|GVAX	Phase I Phase II	Sidney Kimmel Comprehensive Cancer Center, Baltimore, Maryland, USA	NCT03767582	Ongoing
FOLFIRINOX|losartan|nivolumab|radiation: SBRT|procedure: surgery	Phase II	Multicenter	NCT03563248	Ongoing
Niraparib+nivolumab|niraparib+ipilimumab	Phase I Phase II	University of Pennsylvania, Abramson Cancer Center, Philadelphia, Pennsylvania, USA	NCT03404960	Ongoing
Cabiralizumab|nab-paclitaxel|onivyde|nivolumab|fluorouracil|gemcitabine|oxaliplatin|leucovorin| irinotecan hydrochloride	Phase II	Multicenter	NCT03336216	Active, not recruiting
Nivolumab|daratumumab	Phase I Phase II	Multicenter	NCT03098550	Active, not recruiting
FPA008|BMS-936558	Phase I	Multicenter	NCT02526017	Active, not recruiting
BMS-813160|nivolumab|ab-paclitaxel|gemcitabine|5-fluorouracil|leucovorin|irinotecan	Phase I Phase II	Multicenter	NCT03184870	Ongoing

CAR-T, chimeric antigen receptor-expressing T cell; CCR2, chemokine receptor 2; ICI, immune checkpoint inhibition; PD-1, programmed cell death protein 1; PDAC, pancreatic ductal adenocarcinoma; SBRT, stereotactic body radiation; TIL, tumor-infiltrating lymphocyte.

### IL-6 blockade has multicompartmental effects on antitumor immunity

**Figure 3:**
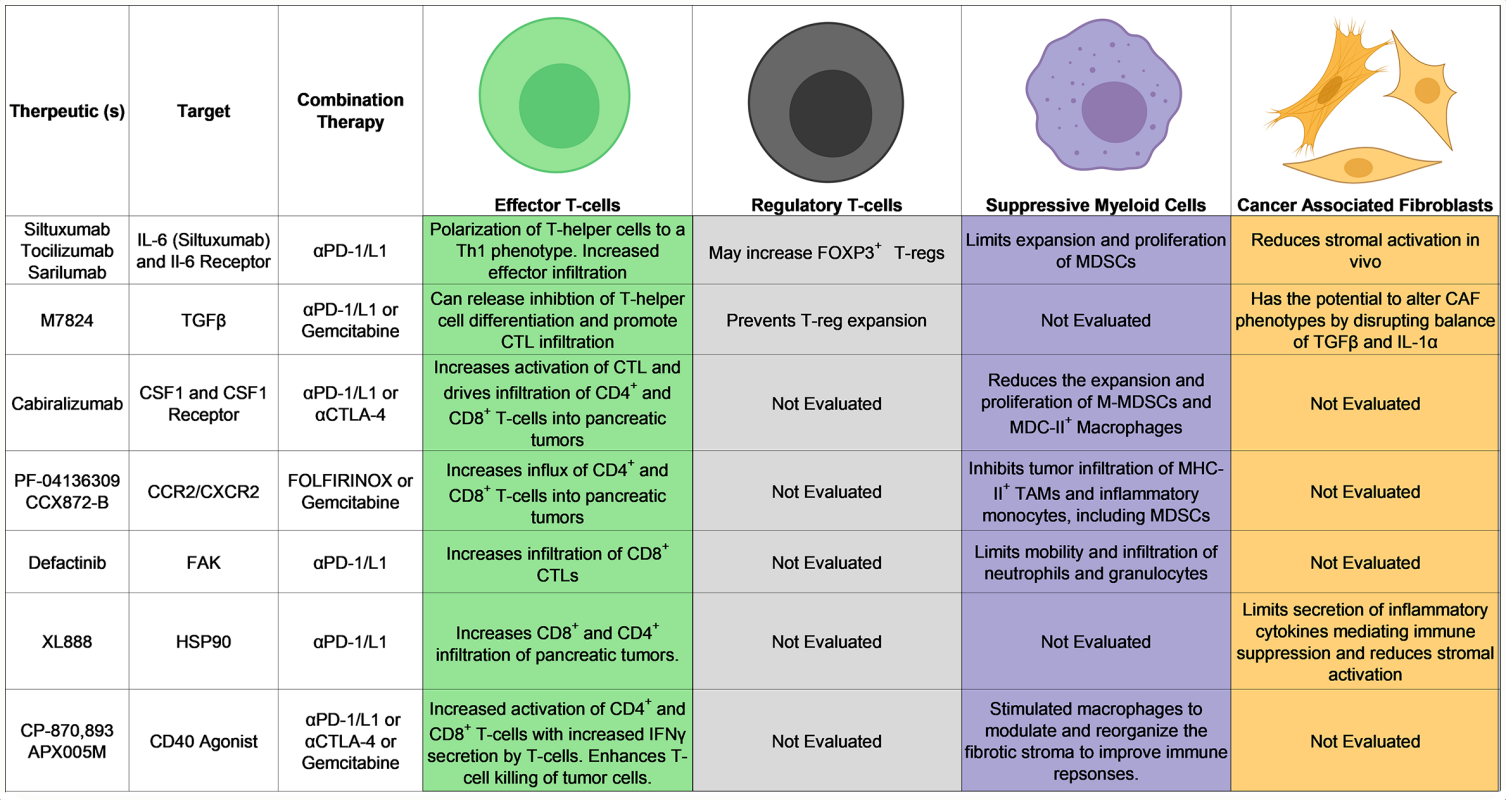
Strategies to reinvigorate immune responses in PDAC target many unique pathways. Shown here are select antibody and small molecule therapeutics combined with ICI in current clinical trials for the treatment of pancreatic cancer. Their respective heterogenous effects on T cells, myeloid cells and CAFs in the TME are highlighted. Of note, many emerging therpaeutic strategies combined with ICI influence multiple cellular populations in the TME of pancreatic tumors. While not thoroughly evaluated, these combination strategies likely influence many other cellular subsets. Putative mechanisms of action are derived from published preclinical data and/or correlative research as part of a clinical trial. ICI, immune checkpoint inhibition; IL, interleukin; TAM, tumor-associated macrophage; TGFβ, transforming growth factor beta; TME, tumor microenvironment; cancer associated fibroblast, CAF; major histocompatability complex-II, MHC-II; CTL, cytotoxic T-lymphocyte.

The complicated cytokine and chemokine milieu of PDAC contributes to immune suppression in various ways. However, certain soluble factors are consistently upregulated by multiple cell subsets in PDAC. IL-6 represents one prominent cytokine consistently present within the PDAC TME. Although this cytokine can be derived from multiple cellular sources, it is transcribed in abundance by human pancreatic stellate cells and drives expansion of myeloid-derived suppressor cells (MDSC) in vitro^80^ (figure 3). The influence of IL-6 on myeloid cells has also been demonstrated in metastatic PDAC, where IL-6 signaling through serum amyloid A1 and A2 promotes myeloid cell recruitment and a prometastatic niche in the liver.^81^ IL-6 can also polarize T-cell responses away from Th1 immunity, characteristic of effective antitumor responses, and regulate balance of Th17 or T regs in a context-dependent manner^82–84^(figure 3). Using murine PDAC models, in vivo blockade of IL-6 enhanced efficacy of anti-PD-L1 antibodies in a CD8^+^ T-cell-dependent manner.^85^ The combination of blocking IL-6 and PD-L1 has since been extended to models of brain, colon, non-small cell lung cancer and others.^86–88^ These preclinical data provide rationale for an ongoing early phase clinical trial at our institution that encompasses a robust series of correlative studies on immune and stromal biomarkers in paired biopsies (NCT04191421). Exciting studies testing IL-6R blockade in combination with chemotherapy (NCT02767557) and immunotherapy/radiotherapy combinations (NCT04258150) have also recently opened at other institutions for patients with metastatic PDAC.

### Restoring balance of T-cell phenotypes by inhibiting TGFβ

Another potent mediator of tumor progression in PDAC is TGFβ, which is present in the stroma of pancreatic tumors.^89 90^ While TGFβ has a powerful influence on malignant cells, this cytokine also influences T-cell function and differentiation (figure 3). With respect to CD4^+^ T cells, TGFβ regulates expansion of cells with Th17 or regulatory phenotypes, depending on the presence of IL-6.^91^ TGFβ also influences the phenotype of CD4^+^ T ells by inhibiting expression of the transcription factors GATA binding protein 3, T-box protein expressed in T cells and subsequent signaling to prevent activation of inflammatory CD4^+^ T cells.^92–94^ More recently, evidence has emerged demonstrating TGFβ signaling in CD8^+^ T cells inhibits both trafficking into tumors and activation of CD8^+^ T cells.^95^ While dual blockade of TGFβ and PD-1 led to tumor regression in preclinical studies, activation of T cells both in the tumor and in the periphery demonstrates the ability of this combination to overcome broad immune suppression in these models.^96^ Currently, a TGFβ ligand trap (M7824) is in clinical trials alongside standard of care gemcitabine for patients with untreated PDAC (NCT03451773).

### Modulating suppressive myeloid cells in PDAC by antibody blockade of CFS1/CSF1R

Investigation of tumor-associated myeloid populations has revealed important mechanisms of suppressed T-cell immunity in PDAC and new targets for therapy. Recently, colony-stimulating factor 1 (CSF1) and its receptor, colony-stimulating factor 1 receptor (CSF1R) have garnered attention in PDAC. The source of CSF1 is likely PCCs themselves, with CSF1R expression localized to the immediately adjacent stroma.^97^ Initial mouse studies blocking CSF1/CSF1R interactions revealed a significant impact on myeloid populations, reducing M-MDSCs and MHC-II expressing macrophages that can directly inhibit T-cell activity in PDAC^97^ (figure 3). Subsequent studies using dual blockade of CSF1R and PD-1 or CTLA-4 profoundly increased both CD4 and CD8 infiltration of tumors and resulted in tumor regression, including complete regression in about 30 percent of mice.^97^ This strategy has now led to a national phase Ia/b clinical trial (NCT02526017) incorporating αPD-1 blockade and cabiralizumab, an antibody against CSF1R.

### CCR2/CXCR2 impacts myeloid populations in PDAC to reinvigorate T-cell responses

Blockade of the chemokine CCR2 has elicited similar, even redundant mechanisms of response to that of CSF1R blockade.^70^ CCR2 has been identified as a crucial mediator of macrophage migration and infiltration into various tumor types.^24 72 73^ While CCR2 inhibitors only modestly impact tumor growth, an impressive antitumor response was mounted in mice treated simultaneously with gemcitabine and CCR2 inhibitor (CCR2i).^70^ Following these promising studies, a clinical trial at Washington University (NCT01413022) and a multicenter trial (NCT02345408) treated advanced/metastatic patients with PDAC with the combination of fluorouracil, leucovorin, irinotecan, and oxaliplatin (FOLFIRINOX) and one of two distinct CCR2i.^10 98^ Both clinical trials elicited excitement with objective responses and prolonged overall survival compared with published results of FOLFIRINOX alone.^10 98^ Correlative studies from these trials revealed decreases in MDSCs, and increased CD4^+^ and CD8^+^ T-cell infiltration in tumor tissue, consistent with data reported in murine models^71^ (figure 3).

### Focal adhesion kinase (FAK) disrupts the mobility of myeloid cells to restore T-cell infiltration into tumors

FAK inhibitors are being explored as a therapeutic option with similar mechanisms of action to CCR2i and CSF1Ri. Interest in FAK has been evident for some time, as the multifunctional protein has been studied for its role in invasion, migration, cell survival and proliferation.^99–103^ Emerging research has exposed a novel role for FAK in mediating immunosuppression.^104 105^ Infiltrating myeloid cells, specifically tumor-associated macrophages and neutrophils, depend on FAK to penetrate tumors with dense ECM.^106 107^ In line with these data, increased FAK activation in human PDAC samples correlates with decreased infiltration of CD8^+^ lymphocytes, increased neutrophils and CD15^+^ granulocytes^104^ (figure 3). These data indicate a correlation between increased FAK activation and cells with an immunosuppressive phenotype in human PDAC tissues. Indeed, FAK inhibition in murine models of PDAC successfully inverted this balance of cytotoxic lymphocytes and suppressive myeloid cells to favor regression of pancreatic tumors^104^ (figure 3). The combination of FAK inhibition with chemotherapy and PD-1 blockade improved survival in mice bearing spontaneous PDAC.^104^ These preclinical results culminated in a phase I clinical trial of gemcitabine, the PD-1 blocking antibody pembrolizumab, and the FAK inhibitor defactinib.^108^ Stable disease was observed in just over 50% of the patients in this trial, with no dose limiting toxicities reported in the dose expansion phase.^108^ This trial is now enrolling patients in a phase II expansion cohort (NCT03727880).

### Inhibiting heat shock protein 90 (HSP90) has a beneficial multicellular impact on the TME

HSP90 is a chaperone protein at the crux of pathways associated with many hallmarks of cancer. These include assisting the folding of proteins such as BRAF, EGFR, fusion proteins like Bcr-Abl and other factors dysregulated in cancer.^109–113^ Immune-associated client proteins of HSP90 such as STAT3, STAT5, and C/EBPε are also of interest, given their involvement in expansion of myeloid cells with suppressive functions.^114–118^ Thus, HSP90 represents a target with a centralized role in many pathways regulating tumor progression while also contributing to a protumorigenic microenvironment. HSP90 inhibitors may also be leveraged for immune modulation.^119–122^ Preclinical studies demonstrate XL888, an HSP90 inhibitor, can elicit efficacy in murine PDAC models when combined with PD-1 blockade and can enhance tumorous infiltration of both CD4^+^ and CD8^+^ cells^123^ (figure 3). It is possible the improved response results from the impact of XL888 on the TME of these tumors. Specially, XL888 can limit activation and inflammatory cytokine secretion from pancreatic tumor-associated fibroblasts (figure 3). These preclinical studies complement an ongoing investigator-initiated phase Ib/II clinical trial (NCT03095781) of XL888 and pembrolizumab (anti-PD-1).

### Reprogramming macrophages to modulate PDAC-associated stroma with CD40 agonists

Given the immunologically cold state of pancreatic tumors, licensing of antigen-presenting cells (APCs) to prime and activate T-cell responses is an area of growing attention. On recognition of antigen on APCs, interaction between CD40 ligand on T cells and CD40 receptor on APCs mediates priming of T cells, described as the transition of T cells to a ‘licensed’ state.^124–126^ This allows for efficient and potent activation of both CD4^+^ and CD8^+^ T cells. The development of CD40 agonists to boost this response was therefore identified as an auspicious therapeutic approach. This strategy was tested in a multicenter phase I dose escalation trial with patients receiving the combination of CD40 agonist and gemcitabine demonstrating improved OS and progression free survival(PFS) versus gemcitabine alone.^127^ Notably, this trial enrolled patients with newly diagnosed, resectable PDAC rather than advanced disease. Priming T-cell responses requires sufficient antigen presentation by APCs; therefore, patients were treated with gemcitabine with CD40 agonist delivered several days after the first dose of gemcitabine.^127^ This approach was used with the hypothesis that chemotherapy would elicit some tumor cell killing to provide APCs in the lymphoid organs with antigen released from dying tumors. These studies raise a particularly important question as to the timing of combination therapy.

Similar results were observed in KPC mice treated with gemcitabine and the CD40 agonist FGK45.^127^ In an interesting mechanistic twist, murine studies in KPC mice revealed macrophages, rather than T cells, as indispensable for FGK45-induced tumor regression, as this CD40 agonist converted the cytokine profile and activity of macrophages to elicit stromal degradation and lysis of pancreatic tumor cells^127^ (figure 3). The ability of CD40 agonists to stimulate stromal modulation and reorganization via macrophage activity suggests other therapeutic modalities could be enhanced without the immunosuppressive stroma characteristic of pancreatic tumors. In melanoma, αCD40 agonists have been combined with CTLA-4 blockade to significantly enhance both T-cell priming and activation with survival benefits for patients (NCT01103635).^128^ Employing a similar strategy, a Phase Ia/b clinical trial (NCT03214250) of gemcitabine plus nab-paclitaxel, the CD40 agonist APX005M, and nivolumab (αPD-1) demonstrated safety and tolerability with promising antitumor activity. Based on encouraging initial data, this combination has proceeded to phase II dose escalation.

### Vaccine and cellular therapies

Very recent advances in technology and understanding of T-cell biology have catalyzed development of vaccine and cellular therapies such as autologous cell transfer and chimeric antigen receptor-expressing T cells (CAR-Ts). There are a number of active clinical trials using vaccine therapies in PDAC with novel strategic targets. These trials include vaccines directing immune responses to tumor-expressed human guanylyl cyclase C and mutated KRAS (NCT04111172 and NCT04117087, respectively), as well as several personalized peptide-based vaccines (NCT03794128 and NCT02600949). The granulocyte-macrophage colony-stimulating factor-based vaccine GVAX is being tested in clinical trials in combination with ICI for PDAC (table 1). Another unique vaccine therapy uses patient-derived, mature DCs, which are directed against mutant KRAS by pulsation with mutant KRAS peptides (NCT03592888). These cells are then used for autologous transfer back into patients to elicit adaptive immune responses.

Adoptive cell therapies have had success in multiple cancer types and are under development for PDAC. These approaches include administration of TIL products and autologous approaches involving T-cell receptor (TCR) or chimeric antigen receptor T cell therapy. While TIL expansion from PDAC tumors can be challenging, CAR-T or TCR-based therapy is governed by identification of appropriate antigens toward which immune responses should be directed.^129–132^ While several tumor-associated antigens continually emerge as targets in pancreatic cancer, the investigation and identification of viable TCR or CAR-T targets is ongoing. Current clinical trials using CAR-T therapy are targeting a variety of antigens, including carcinoembryonic antigen, mesothelin, CD133, CD70, human epidermal growth factor receptor 2, epithelial cell adhesion molecule and many others, which we have previously reviewed.^133^ Additionally, prostate stem-cell antigen (PSCA) targeting CAR-T therapy is currently in clinical trials (NCT02744287) and has shown promising interim results. The broad heterogeneity of antigen presentation by cells within solid tumors makes CAR-T design extremely challenging. An innovative approach by Marker Therapeutics is testing a T-cell product directed against up to five tumor-associated antigens simultaneously, which is currently in phase I/II clinical trials (NCT03192462). Additionally, with the discovery of T-cell subsets with high potency against tumors, the field of cellular therapy for PDAC is rapidly expanding. Further, these cellular therapies will encompass significant advances in innovation, such as cytokine, antibody or chemokine receptor engineering that may be advantageous for notoriously hard-to-treat diseases such as PDAC.^134^

## Conclusions

Our knowledge of immune suppression in PDAC is quickly expanding and providing new therapeutic opportunities in the realm of this disease. The field is now rich with numerous emerging combination therapies that elicit effects across multiple cellular components of the TME to promote antitumor T cell-mediated immune responses (figure 3). Rapidly growing technology in the field of antibodies, small molecules, vaccines, gene therapy and engineered T cells show promise for pancreatic cancer, among other aggressive diseases. Furthermore, our continually improving ability to dissect molecular and genetic mechanisms mediating tumor progression provides opportunity for individualized targeted and immune therapy. With careful attention to complex cell–cell interactions in the PDAC TME, we can certainly improve our ability to invigorate T-cell responses to these recalcitrant tumors.
